# Horst Aspöck, encyclopedist and entomologist extraordinaire – a personal appreciation

**DOI:** 10.3897/zookeys.555.7410

**Published:** 2016-01-20

**Authors:** Michael Ohl

**Affiliations:** 1 Museum für Naturkunde, Leibniz-Institut fuer Evolutions- und Biodiversitaetsforschung, Invalidenstr. 43, D-10115 Berlin, Germany

**Keywords:** Biography, Neuropterida, Raphidioptera, Neuroptera, Megaloptera, history of science

## Abstract

The paper provides an overview of the life and work of Prof. Dr. Horst Aspöck, the doyen of neuropterology, on the occasion of his 75^th^ birthday. It particularly emphasizes his outstanding contributions to the development of neuropterology since the 1960s.

## Introduction

This is a note on one of the most outstanding and productive entomologists of our time. It is not meant to be an exhaustive account of Horst Aspöck’s career and achievements over the 75 years of his life so far. My knowledge of Horst is somewhat limited to his entomological, historical and linguistic activities and stems mostly from my professional and personal relationship with him over the last ten years. Numerous biographical essays on Horst have been published on various occasions and from different perspectives, and it is certainly advisable to read them for a more complete picture of Horst than I can provide here (e.g., Auer 1999, Knapp 2004, Gusenleitner 2004, Thaler 2004, Christian 2009). These authors have covered some aspects of his life, which I will not repeat here, including Horst’s extensive fieldwork (e.g. Rausch and Rausch 2004), his role as an academic teacher, his varied roles in scientific societies, and finally the numerous awards and prizes he received. Here I will briefly summarize his life and career, with information derived from these published biographies, and give my personal impressions of his influence on neuropterology and entomology in general, and finally on my personal relationship to him.

Writing such a paper about Horst’s personal life and scientific work with a significant biographical and entomological focus is a challenge not only because of the sheer amount of potentially relevant material, but also because Horst’s academic work in entomology is inextricably intermingled with the life, scientific work and career of his wife Ulrike. In 1964, they began to work and publish together on Neuropterida, and most of their publications on Neuropterida published since then are coauthored by the Aspöcks together, often with additional coauthors. Trying to carve out Horst’s many achievements is often hardly possible, since most of them were actually Horst’s and Ulrike’s combined achievements. However, since this paper is written in honor of Horst’s 75^th^ birthday, I try to follow his personal tracks over time, but I am aware that Ulrike’s and his life and work are too closely connected to do that consistently. Horst himself has worked out their close and intimate life-long collaboration in a paper published on the occasion of her 70^th^ birthday (H. Aspöck 2012).

## Horst’s life and career (Figs 1–7)

Horst was born on 21 July 1939 in Budweis (České Budějovice) in former Czechoslovakian and now Czech Bohemia. At the time of his birth, Czechoslovakia has already been annexed by the German Reich, and as late as early 1945, the World War also arrived in Budweis. Until May 1945, Bohemia and Moravia were the very last war zones of World War II in Europe, until the German Armed Forces capitulated on 8 May 1945. Local inhabitants of German origin had to suffer severe retaliations, and according to the Potsdam Agreement from 1945, all Germans were expelled from the Sudetenland region. Horst’s mother, Maria (“Manka”) Knapp, was of Austrian nationality, because she was married to the Austrian-born Fritz Aspöck, from whom she had already divorced in 1941. In the fall of 1945, the Aspöck family moved to Linz in the Austrian state of Upper Austria.

Horst lived in Linz with his family until 1957, when he enrolled at the University of Innsbruck to study Biology with a Zoology major. On the 12^th^ July 1962, he received his Ph.D. from the University of Innsbruck with a thesis on the toxicological characteristics of carbamates. Shortly after, Horst became a student assistant at the Institute of Hygiene of the University of Vienna, and he continued working at this very same university until his retirement in 2004. In January 1963, he became research assistant, and in 1966, he was commissioned to establish and lead a new department of medical parasitology at the Institute of Hygiene of the University of Vienna. In 1970, he received his habilitation and was promoted to “Universitätsdozent” for medical parasitology. In 1977, Horst was promoted to extraordinary Professor and in 2000 to full university professor for medical parasitology. He worked as the head of the department of medical parasitology at the same institute, until he retired in September 2004. Since retirement he has continued duties in academic teaching and research and in the supervision of students. Over the years, he has received numerous awards and became honorary or corresponding member in several national and international societies. He is a member of the Nationale Akademie der Wissenschaften Leopoldina since 2000 and a member of the Human-Rights-Committee of the Leopoldina since 2003.

In 1963, Horst married Ulrike Pirklbauer, who is better known as Ulrike Aspöck in entomology. They have one son, Christoph, born in 1965.

**Figures 1–7.**
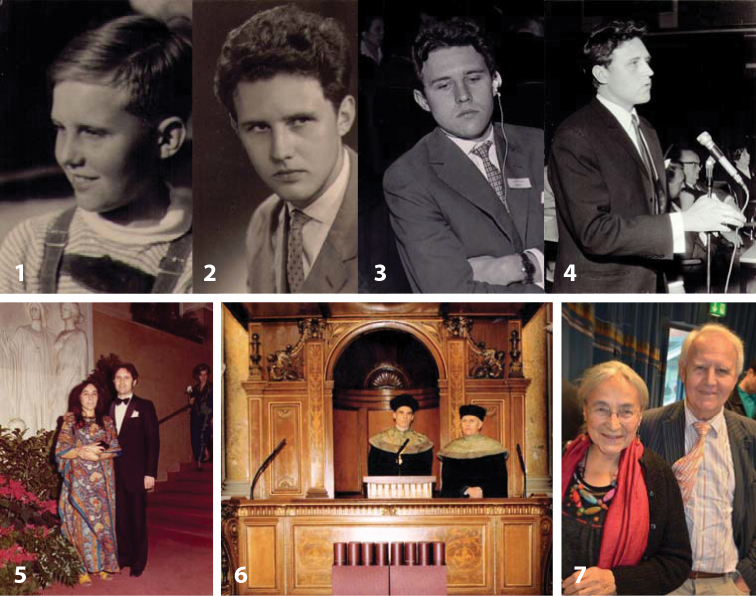
Horst Aspöck, developmental stages and as a gentleman and professor. **1** around 1950 in Linz, Upper Austria **2** In 1961, passport photo **3** In 1963, Prague, Czechoslovakia, during a symposium on “Theoretical questions of Natural Focis of Diseases” **4** In 1970, Igls near Innsbruck, Austria, during a conference of the German-speaking society for tropical medicine **5** In 1973, in Vienna, Austria, as enthusiastic dancers on a ball at the Wiener Konzerthaus **6** 2004, in the Festsaal of the University of Vienna. With vice rector Hans Geord Eichler (left), during the first PhD defense of the Medical University Vienna (previously Medical Faculty of the University Vienna), held in Latin language, as still mandatory in Austria **7** In 2014, in Linz, Austria, with Ulrike, during the 81^st^ International Entomologist’s Conference. All photos from the Aspöck photo archive.

## Horst’s way to Entomology and to Neuroptera

**Figures 8–14.**
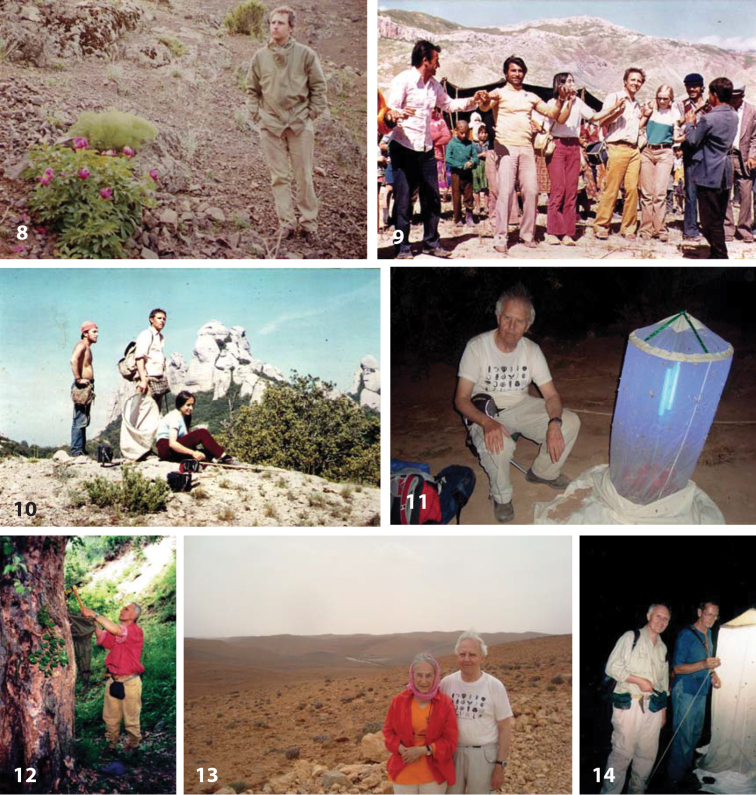
Horst Aspöck in the field. **8** In 1975, Anatolia, Turkey. During a collecting trip in the Pontus-mountains to collect Raphidioptera (photo by Hubert Rausch) **9** In 1975, Doĝubayazit, Anatolia, Turkey. Horst, flanked by Ulrike Aspöck (left) and Renate Rausch, dancing with locals **10** In 1971, Catalonia, Spain. From left: Ernst Hüttinger, Horst, Ulrike **11** In May 2014, Anti-Atlas, Morocco, Horst night-collecting **12** In 1995, Talasskaya Oblast, Talasskiy Alatau, Kyrgyztan. Horst collecting larvae of Raphidioptera **13** In 2014, Anti-Atlas, between Tafraoute and the Igmir Oasis, with Ulrike **14** In 2000, Mae Hong Son Pai Province, Thailand, night-collecting with the Austrian entomologist Hans Malicky. All photos except for photo 8 from the Aspöck photo archive.

As early as 1952, at the age of 13, Horst became a member of the “Entomologische Arbeitsgemeinschaft” at the “Oberösterreichisches Landesmuseum”. Getting in close contact with experienced and enthusiastic entomologists was quite influential, and only four years later, in 1956, he joined an entomological field trip to Istria. He regularly gave private lessons in order to cover the costs of his entomological activities. From the very early beginning, he also showed a disposition to classical languages and to linguistic subtleties, which largely influenced his writing and his taxonomic work until today. During his university studies in Innsbruck, Horst continued his entomological activities, amongst others by temporary work at the “Commonwealth Institute of Biological Control” in Delémont, Switzerland. Although he had already developed some interest in lacewings during the field trip to Istria, he started to work systematically on Neuroptera in 1960 (H. Aspöck 2003a). In 1962, Horst began to study snakeflies from Greece and Anatolia from the personal collections of Josef Klimesch and Franz Ressl, two Austrian entomologists, and he soon realized that the diversity of Raphidioptera in the eastern Mediterranean, particularly in Greece, was much larger than expected from the literature and in contrast to the overall uniform external morphology of snakeflies. Right from the beginning of his developing fascination on Neuroptera, he started to publish his observations and results. In 1962 alone, Horst published five papers (see Gusenleitner 2004, 2009, 2014, for bibliographies of Horst’s work), two about the biological effect of carbamates as a result of his Ph.D. thesis, but three papers already on Neuroptera. Two of them dealt with the taxonomy of Hemerobiidae, but one was entitled “Gedanken zur Erforschung der Neuropterenfauna Österreichs” (Thoughts on the exploration of the neuropteran fauna of Austria) (H. Aspöck 1962). It is remarkable and quite symptomatic, that one of his very first publications, which Horst has published on Neuropterida, was an overview on the state-of-the-art of the knowledge of Austrian Neuropterida. Retrospectively, this publication can be seen as the very early announcement of his personal research program on Neuroptera for the following decades. Even more, it is a very early example of Horst’s tendency to keep the general picture in mind, even when working on small taxonomic projects. In this small paper from 1962, he concluded that the knowledge of the Austrian fauna of Neuropterida is incomplete, and he spent most of the text to present the reader an overview on the diversity of Austrian lacewings, how the various families can be recognized and where they can be found. Consequently, a brief note was added, where Horst asked members of the “Arbeitsgemeinschaft Österreichischer Entomologen” to send him any neuropteran they would collect in exchange for other insects from Central and Southern Europe. In 1963, Horst and Ulrike not only married, but they also began a life-long and fruitful collaboration on Neuropterida, particularly on Raphidioptera. The very first results were *Raphidia ulrikae*, a new Central European snakefly species, which Horst dedicated to his wife (H. Aspöck 1964), and the first publication coauthored by the Aspöcks on new species of *Raphidia* (H. Aspöck and U. Aspöck 1964). In the following decades, they would not only publish, but also travel together to numerous countries on all continents to collect Neuropterida (Figs 8–14, see also Rausch and Rausch 2004).

## Entomology

I have only limited knowledge about Horst’s research activities in medical parasitology, but these have been presented and cherished in several papers by more competent authors, including Auer (1999), Flamm (2007, 2012) and publications in the Festschrift “Entomologie und Parasitologie”, edited by Ulrike Aspöck (2004), on the occasion of Horst’s 65^th^ birthday. The “duality” (Thaler 2004) of contributing significantly to two different scientific fields in two different scientific disciplines is more than unusual and exemplifies probably more than anything else the broad and general perspective of Horst’s character. I will here concentrate on his entomological work, but also on an additional “side-interest” in the history of science.

Horst is unusually productive, and the “Aspöck-and-Aspöck” publication list comprises 739 publications until 2014, including more than ten books and numerous book chapters (Gusenleitner 2004, 2009, 2014). In the last half a century, almost exactly 50 years after his first publication on Neuropterida, the Aspöcks are more than anybody else at the forefront of neuropterid research, and it is, thus, not surprising that they have authored a large number of chapters on Neuropterida in many contemporary textbooks on zoology, entomology and in several catalogs. Prominent examples are the chapter in “The Insects of Australia” (H. Aspöck and U. Aspöck 1991), the four chapters on neuropterida, Neuroptera, Raphidioptera and Megaloptera in the second edition of the German written “Lehrbuch der Speziellen Zoologie” (U. Aspöck and H. Aspöck 2003a-d), and their catalog of the Neuropterida of the Western Palearctic (H. Aspöck et al. 2001).

Some of the publications, particularly some books, are clearly milestones in their field and proved to be influential for generations of neuropterologists. Two particularly outstanding examples are the “big green books”, as a reference to the color of their book cover, “Die Neuropteren Europas” (The Neuropterans of Europe, H. Aspöck et al. 1980) and “Die Raphidopteren der Erde” (The Raphidiopterans of the World, H. Aspöck et al. 1991). Both of them consist of two volumes and they have scholarly summarized the available knowledge on these two orders as available at that time. They are accompanied by 900 (Neuroptera) and 1300 (Raphidioptera) line drawings and several distribution maps, tables and other kinds of images. Although written in German, both monographs received a worldwide distribution and appreciation, and they are still among the most significant book length monographs on Neuroptera and Raphidioptera ever written. They can clearly be called modern classics in neuropterology.

Although the Aspöcks are especially influential in Raphidioptera research, they have published intensively on virtually all families in Neuroptera, with a specific interest in Berothidae (e.g., U. Aspöck et al. 2013). The majority of publications are on the taxonomy and phylogeny of Neuropterida, although, when feasible, information on the behavior has also been published, including review papers like H. Aspöck (2002a). With the increasing worldwide overview on the diversity and distribution of Neuropterida, particularly Raphidioptera, specific distribution patterns in space and time became obvious, and Horst has published on this historical biogeography intensively. One of the obvious peculiarities of the Raphidioptera is their strictly northern hemisphere distribution, which deserves explanation. One of the more recent publications on global distribution patterns in Raphidioptera is H. Aspöck and U. Aspöck (2004b).

Horst has published intensively in collaboration with his wife Ulrike, and the enormous number of publications with both Aspöcks as authors is remarkable. Only between 2009 and 2014, 71 of a total of 88 publications have been co-authored by both of them (Gusenleitner 2014). In the same period of time, Horst has published with an incredible number of more than 370 different co-authors. Although this high number is biased due to a single publication on a phylogenomic analysis of insect evolution with almost 100 co-authors (Misof et al. 2014), only a relatively small portion of the 88 publications have been published with Horst as sole author. This large number of co-authored publications clearly emphasizes Horst’s understanding of science as a culture of networking and exchanging information, data and material.

The multi-authored publication by Misof et al. (2014) is an indicator of another remarkable character of Horst. In his parasitological research, molecular methods have been standard techniques for quite a long time, particularly when studying viruses and the wide variety of pathogenic organisms. For quite a few decades, Horst has intensively used molecular methods in medical research, usually in close collaboration with competent colleagues and students. In contrast, by far the majority of his entomological activities and publications are based on a careful and exhaustive study of morphological characters in the frame of a comparative approach. However, with the increasing importance of molecular methods in systematic entomologies, he (and his wife Ulrike Aspöck) immediately realized the benefits of the molecular approach. Together they published a first outline of the importance of molecular studies in Neuropterida (U. Aspöck et al. 2003), which was followed by publications on the molecular phylogeny of the Neuroptera (Haring and U. Aspöck 2004, without Horst) and the Raphidioptera (Haring et al. 2011), and a few smaller contributions largely based on the dataset by Haring et al. (2011) with discussions of the implication of the molecular analysis on the biogeography of snakeflies. More recently, Horst and Ulrike contributed to a large genomic project lead by Bernard Misof (Museum Alexander Koenig), which resulted in a series of conference lectures and by two prominent publications on the phylogeny of insects (Peters et al. 2014, Misof et al. 2014). There is more to come soon.

## History, language, and catalogs

Horst has always grounded his empirical work on a fundamental understanding of the historical development of the discipline. He has published repeatedly on the history of neuropterology, including detailed and often personal appreciations of deceased colleagues, like the almost 90-pages-obituary for Herbert Hölzel (H. Aspöck 2009) with the telling title “Ein sehr persönlicher Nachruf und ein Stück Geschichte der Neuropterologie” (A very personal obituary and a piece of history of neuropterology). Over the years, Horst has published more than 60 biographical papers, most of which on colleagues in medical parasitology and in neuropterology (Gusenleitner 2004, 2009, 2014). Some of these publications are of broader significance, like a monographic treatment on all authors who have published taxonomic names in Raphidioptera (H. Aspöck and U. Aspöck 2014a).

Besides the many obituaries and personal biographies, Horst has intensively published on the history of neuropterology in Austria (H. Aspöck 1984) and in the German speaking countries (H. Aspöck and U. Aspöck 2010), on the history of the “International Association of Neuropterologists” (H. Aspöck 2010) (Fig. 20), and on the history of the “Österreichische Entomologische Gesellschaft” (H. Aspöck 2003b). His broad knowledge of the historical literature is best reflected by a series of beautifully illustrated, detailed publications of early descriptions and illustrations of Raphidioptera (H. Aspöck 1998), Mantispidae (H. Aspöck 1999) and Osmylidae (H. Aspöck 2002b).

**Figures 15–20.**
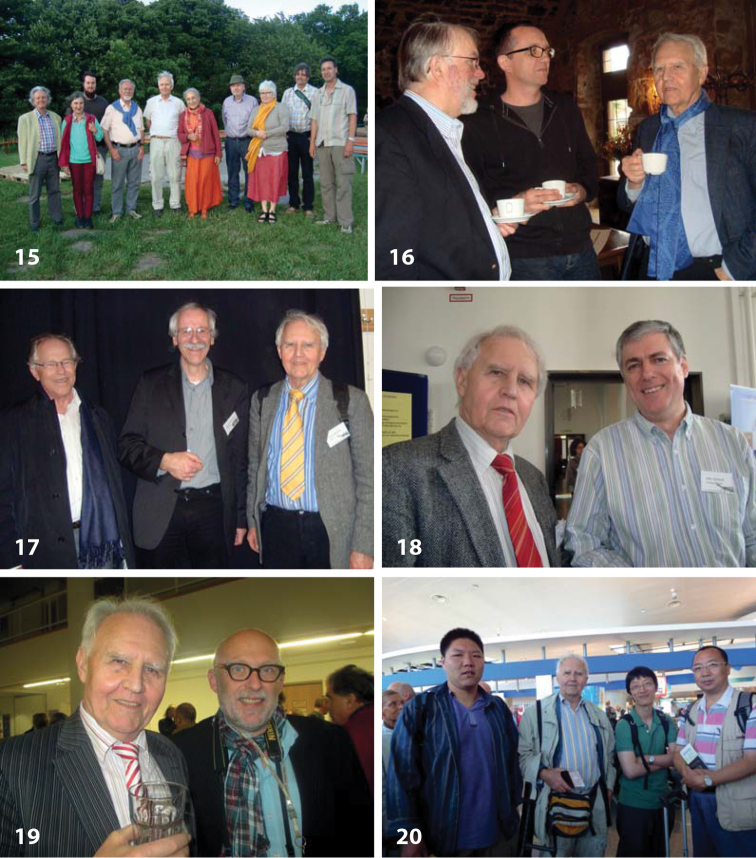
Horst Aspöck among colleagues and friends. **15** In 2013, group photo of the 13^th^ workshop of the German-speaking neuropterologists on Schwanberg Castle, Bavaria, Germany. From left: Johannes Gepp, Melitta Fuchs, Lukas Kirschey, Karl Meissner, Horst and Ulrike, Wilfried Wichard and his wife, Steffen Potel, and Axel Gruppe **16** In 2014, same meeting, with Hubert Rausch and Michael Ohl (from left) **17** In 2011, Berlin, Germany. Annul meeting of the “Deutsche Gesellschaft für allgemeine und angewandte Entomologie (DGaaE), with Ernst Joachim Tröger and Rainer Willmann (from left) **18** In 2011, same conference, with John Oswald (right) **19** In 2014, Vienna, Austria, with Fritz Gusenleitner (right) **20** In 2011, Ponta Delgada, Azores, Portugal. XI International Symposium on Neuropterology, with a group of Chinese neuropterologists. From left: Yongjie Wang, Horst, Xingyue Liu, and Dong Ren.

Horst had a profound education in classical languages, particularly in Latin, and he has developed a good sense for the subtleties of his own mother tongue. In his German publications, Horst develops a complex language, which is not only scientifically accurate and detailed, but also a delight to read. It seems to be logical, at least from my perspective, that Horst has thought about the linguistic background in general and for his science since very early on. This can best seen in the wide variety of taxon names he proposed in Neuropterida, which clearly exhibit a deep understanding of classic languages, combined with careful observation and a sense of humor and fantasy. The etymological origin of all names in Raphidioptera published by him and Ulrike has been presented in a long paper on the etymology of all names in Raphidioptera (H. Aspöck and U. Aspöck 2013).

## My personal appreciation

In the title of this contribution, Horst has been called an “encyclopedist”, and I am well aware that this term has a somewhat old-fashioned connotation. However, his approach in a sense of broadly compiling data and information is encyclopedic both in a historical and a completely modern sense. “Encyclopedist” is truly an historical word, and it derives from a time at least back to the 18th century, when formal biological nomenclature originated from the work of a true encyclopedist, Carl Linnaeus. Since 1758, the year of the publication of the 10th edition of Linnaeus’ “Systema Naturae”, which has been assigned the birth of zoological nomenclature, taxonomists have struggled hard to catalog and register all life on Earth. In a sense, the backbone of Horst’s work lies in the direct tradition of the “Linnaean enterprise” of a global register of all living forms (Wilson 2005). In the 18th century, Linnaeus was inspired by the “dream of completeness” (Frangsmyr et al. 1990), but even at his time, he and his disciples and contemporaries suffered from what the historian Staffan Müller-Wille paraphrased as “information overload” (Müller-Wille and Charmantier 2012). Taxonomic systems and methods were developed to organize and control the ever increasing amount of available information. Linnaeus’ “dream of completeness” was based on a dramatic underestimation of the true amount of the global diversity, and the “Linnaean enterprise” as a full mapping of the Earth’s biodiversity is still far from being completed. It has been extensively discussed, which strategies and technologies are needed “for a comprehensive mission to explore and document Earth’s species” (Wheeler et al. 2012), and Horst and Ulrike have decided to concentrate on a relatively small group of the hyper-diverse insects, which guarantee to approach completeness as close as possible. Neuroptera and Raphidioptera seem to be the perfect target. The diversity is sufficiently large for a life-long and challenging research agenda, but small enough to cover a large, if not the largest portion of the global diversity within a career.

This is how I perceived Horst and Ulrike Aspöck, when I first met Ulrike in mid-1994 in the Naturhistorisches Museum in Vienna. Shortly before I had started to work on my dissertation on apoid wasp taxonomy and phylogeny under the supervision of Rainer Willmann at the University of Göttingen, Germany (Fig. 17). Willmann had sporadically published on Neuroptera before, although he was largely concentrating on Mecoptera. In the early 1990s, when I visited the museum in Vienna for the first time, he had published a few papers on the phylogeny of Mantispidae (Willmann 1990, 1994). Based on morphological characters and the fossil record, he argued in favor of a sister-group relationship between the Mantispidae and Rhachiberothidae, with raptorial forelegs as one of their synapomorphies. This was in contrast to the Aspöcks’ assumption of a monophyletic Berothidae + Rhachiberothidae, with the Mantispidae being their sister-group. This hypothesis has been confirmed in numerous analyses since then (e.g., U. Aspöck and Mansell 1994). With the scientific conflict between Rainer Willmann and the Aspöcks in mind, being a Ph. D. student of Willmann, and well aware of the extraordinary standing of the Aspöcks in entomology in general and particularly in Neuropterida, I was quite nervously looking forward to meeting Ulrike for the first time. She proved to be exceptionally hospitable and intrigued to learn about me and my scientific project. I felt warmly welcomed by her even as a young Ph. D. student, and this combination of scholarly curiosity, kindness and openness is certainly a typical character of Horst and Ulrike, which I have had the pleasure to experience whenever I meet them.

In the following years, I met Horst on various occasions on entomological meetings and conferences, and his personal appearance always inspired me respect. He was, and still is, always critically listening to presentations, even from students giving their first talk, and he very often asks the very first question during the discussion. We first got into a closer contact, when I started to work on Mantispidae within Neuroptera in the late 1990. I was appointed as curator for Neuropterida at the Museum für Naturkunde in Berlin in 1997, and soon after I decided to complement my interest in Hymenoptera by setting up a new, initially smaller project line on Neuroptera. Mantispidae was an attractive group, not only because of its intriguing morphology and behavior, but also because the diversity was limited, with about 350 valid species known, in contrast to the more than 10,000 species of apoid wasps I was confronted with for many years. Even more, after reading through the relevant literature and after talking to the Aspöcks and other neuropterologists, it became clear that at that time, there was nobody seriously working on the taxonomy and systematics of the Mantispidae with a global perspective. As a first step, I started to compile a personal catalog of all taxonomic names, which can be assigned to Mantispidae. I talked to Horst and Ulrike Aspöck frequently about the catalog, and Horst was always curious to learn about the progress I was making. My conversation with him about Mantispidae was usually accompanied by some kind of “examination” of my knowledge mostly about historical literature on Mantispidae, and in the first years, I was always nervous not to reveal too many painful and embarrassing knowledge gaps. However, I not only learned a lot from talking to Horst, but I very much enjoyed discussing with him topics of mutual interests. My “personal mantispid catalog” was finally published a few years later (Ohl 2004).

Over the last decade, I met Horst regularly on the meetings of the “Arbeitskreis Neuropteren” (http://www.dgaae.de/index.php/neuroptera.html), formerly the “Arbeitstreffen deutschprachiger Neuropterologen” (Figs 15–16). This has been established as a study group within the “Deutsche Gesellschaft für Allgemeine und Angewandte Entomologie”, the largest German entomological society. The meetings of the “Arbeitskreis” take place in the “Kloster Schloss Schwanberg” in the Bavarian area of Lower Franconia, and it is a very stimulating place. The “Arbeitskreis” is a rather small group, organized by Axel Gruppe (Fig. 15), Freising, Munich, and Horst and Ulrike clearly play a central role in this small scientific community. For me, this is an especially pleasant opportunity to talk extensively to Horst about plans, ideas and particularly Neuroptera. Since families are also welcomed on these meetings, I frequently take my own family with me. Horst, now himself being a grandfather, was always very interested in my wife Daniela and my children Mattes, Merle and Mina, particularly when he realized that my son Mattes is already in the process of becoming an enthusiastic naturalist, with serious interests in spider taxonomy and the Latin language.

Besides Neuroptera, Horst and I have a great deal in common, and two particular interests we share are the linguistics and etymology of taxonomic names and the history of entomology, not to speak of neuropterology. Ulrike’s and his monographic treatment of the etymology of names in Raphidioptera (H. Aspöck and U. Aspöck 2013) and my own popular science book on the culture of naming in natural history (Ohl 2015) developed simultaneously, though coincidentally. Horst’s interests in the many aspects of the history of entomology, neuropterology in general, neuropterology in Austria and many other aspects are best demonstrated by the extraordinary private Aspöck library. Horst is an enthusiastic collector of historical books in natural history, and their personal book collection is far beyond any other personal library I know. Neuropterological literature is predominant, but there are many more historical books, which make their personal book and reprint collection an impressive library. Horst was lucky to start collecting rare books at a time, when the international book market was not dominated by the internet, so that he could buy many rare books for reasonable prices, which would be hardly affordable today. My own book collection is significantly smaller, but we enjoy sharing our knowledge and interest about historical books whenever possible.

The celebration of Horst’s 75^th^ birthday is the perfect opportunity to look back to his many accomplishments. It is obvious from the above that Horst has a fearsome intellect and exhibits boundless dedication. He is working hard in pursuit of his exceedingly broad passion, and he is not slowing down significantly. He is a real gentleman, both personally and as a scientist, and he is very thoughtful of the needs of friends and colleagues. He is much sought as a collaborator, first because of his broad knowledge in so many fields, but also because he has the focus and concentration to bring projects to a success. I admire his continuing energy, his productivity and his drive. He is setting a high standard for the younger generation, including me, and although I am not always sure that I am able to meet these standards myself, I can call myself very fortunate to benefit from extensive interactions with Horst in so many respects. We are all looking forward to much more to come.
